# Editorial: Methods in general cardiovascular medicine

**DOI:** 10.3389/fcvm.2023.1277373

**Published:** 2023-08-29

**Authors:** Serafino Fazio, Silvia Marchiano, Coen van Solingen, Jin Li, Qingchun Zeng

**Affiliations:** ^1^ Federico II University Hospital, Naples, Italy; ^2^ University of Washington, Seattle, WA, United States; ^3^ New York University, New York City, NY, United States; ^4^ Shanghai University, Shanghai, China; ^5^ Southern Medical University, Guangzhou, China

**Keywords:** methods, protocols, cardiac function, atrial fibrillation, arterial stiffness

**Editorial on the Research Topic** Methods in general cardiovascular medicine

Cardiovascular diseases are the leading cause of death worldwide. The advancement of scientific knowledge and clinical practice in general cardiovascular medicine relies on innovative and groundbreaking approaches. The Research Topic *Methods in General Cardiovascular Medicine* has collected 11 valid manuscripts that will help researchers and clinicians to design, conduct, evaluate and apply the best evidence-based interventions to prevent, diagnose and treat cardiovascular diseases.

This editorial highlights the important aspects discussed in the published manuscripts of the Topic.

Pironti, in his review paper, carries out an in-depth analysis of the methods used to perform an effective evaluation of the systolic and diastolic cardiac function in mice, including pitfalls and alternative solutions.

The study by Li and Zheng reviewed the methods currently available for the evaluation of the sympathetic heart tone in clinic and research, explaining the principles underlying these methods. The authors conclude that all methods for assessing cardiac sympathetic activity are based on the anatomy and physiology of the heart, and, particularly, on the innervation and sympathetic regulation of the heart. For this reason, technological advances, the overlapping of disciplines and an improved understanding of the sympathetic innervation and regulation of the heart will certainly promote the development of new, more accurate methods of assessing cardiac sympathetic activity.

In another study published in this Research Topic, Lin et al. used Korotkoff sounds to measure blood pressure, with the application of Deep Learning (DL); they suggested that the application of DL to Korotkoff tones might be used for the early diagnosis of heart failure (HF). Considering the high mortality and morbidity of HF, and the importance of an early diagnosis, this novel application of Korotkoff sounds could be very useful, particularly in some rural areas, or in areas where advanced medical instruments are not easily accessible.

The following three studies of this Research Topic are Original Research studies. The study by You et al. retrospectively compares the results obtained with three catheter ablation methods in patients with episodes of paroxysmal atrial fibrillation (PAF). Their results showed no statistically significant differences in PAF recurrence among the three methods used, (radiofrequency, cryoablation and a combination of the two), suggesting that the type of ablative procedure is not a determinant in AF recurrence. They also found that only left atrial appendage emptying velocity resulted to be an independent predictor of PAF recurrence.

Zhang et al. in their study describe a method to establish the threshold values of Arterial Velocity-pulse index (AVI) and Arterial Pressure-Volume index (API), measured by a brachial cuff, indicative of arterial stiffness. The study demonstrated that AVI and API can be used to perform a preliminary screening of increased arterial stiffness. Because of the direct correlation between increased arterial stiffness and the risk of cardiovascular events, this simple method allows the early identification of subjects in the general population more at risk of cardiovascular events, enabling a prompt preventive treatment.

The study by Wang et al. aimed to evaluate the reliability, convergent and known-groups validity of protocols that used sit-to-stand tests (STS) to estimate the level of risk for cardiovascular events in patients with coronary artery disease (CAD). The conclusions of the study were that all three STS tests (Five times STS test, 30-s STS test, 1-min STS test) had good test-retest reliability, convergent and known-groups validity, and that these tests can distinguish low-risk CAD patients from high-risk ones of cardiovascular events.

The other five manuscripts of this Research Topic describe the protocols used in their respective studies to obtain reproducible results.

In their manuscript, Yoon et al. describe a study protocol for a randomized controlled trial aimed to verify if, applying smartphone applications and mobile health platforms, was possible to improve the adherence to treatment with Rivaroxaban, a non-vitamin k antagonist oral anticoagulant (NOACs).

Bruch et al. in their manuscript, describe the protocol of a study to determine which factors (including comorbidities and social-economic status) influence the implementation of digital prevention of arterial hypertension, particularly in the most remote and sparsely populated areas, and thus improve cardiovascular outcomes.

In *Early vascular healing after neXt-generation drug-eluting stent implantation in Patients with non-ST Elevation acute Coronary syndrome based on optical coherence Tomography guidance and evaluation (EXPECT): study protocol for a randomized controlled trial* by Zhu et al. the authors assess early vascular healing after next-generation drug-eluting stents (DES) implantation in patients with non-ST elevation acute coronary syndrome (NST-ACS) guided and evaluated by optical coherence tomography (OCT). The study includes 60 patients randomized at 1:1:1 ratio to OCT guide percutaneous coronary intervention (PCI). The primary endpoint of the study is to verify the rate of vascular healing at the stent level in the three study groups. The data from the study are expected to provide new insights into vessel wall healing in an NST-ACS population following next-generation DES implantation, underscore the value of OCT in speeding vessel healing, and evaluate the possibility of early discontinuation of dual antiplatelet therapy in this population.

Gao et al. in their manuscript, describe a study protocol for an innovative off-the-shelf aortic endograft system for the treatment of juxta renal abdominal aortic aneurisms. The primary efficacy endpoints will be the frequency of immediate technical success of the implant and the number of reoperations within 12 months of the procedure. There will also be a primary safety endpoint: the frequency of major adverse events within 30 days of implant. The authors also describe the strengths and limitations of the study protocol. In particular, the major limitation of this study is the lack of a control group.

Finally, the manuscript of Ullrich et al. describes the protocol to verify whether an improvement in vascular resistance of coronary artery can be achieved by increasing retrograde pressure in the coronary sinus (CS). The changes of coronary vascular resistance could improve the symptoms of microvascular angina, making the pathophysiology of the disease better understood, and indicating new therapeutic perspectives. The study design will be randomized and crossover, with patients in whom coronary hemodynamics will be analyzed during partial balloon occlusion in the CS, and patients in whom coronary hemodynamics will be measured with a completely deflated balloon. The primary endpoint of the study will be the change in the hemodynamically obtained coronary resistance index following acute changes in CS pressure.

